# Time- and Concentration-Dependent Adverse Effects of Paclitaxel on Non-Neuronal Cells in Rat Primary Dorsal Root Ganglia

**DOI:** 10.3390/toxics11070581

**Published:** 2023-07-04

**Authors:** Amira Elfarnawany, Faramarz Dehghani

**Affiliations:** 1 Department of Anatomy and Cell Biology, Medical Faculty, Martin Luther University Halle-Wittenberg, Grosse Steinstrasse 52, 06108 Halle (Saale), Germany; amira.elfarnawany@science.tanta.edu.eg; 2 Zoology Department, Faculty of Science, Tanta University, Tanta 31527, Egypt

**Keywords:** peripheral neuropathy, DRG non-neuronal cells, paclitaxel, MTT assay, LDH assay, BrdU assay

## Abstract

Paclitaxel is a chemotherapeutic agent used to treat a wide range of malignant tumors. Although it has anti-tumoral properties, paclitaxel also shows significant adverse effects on the peripheral nervous system, causing peripheral neuropathy. Paclitaxel has previously been shown to exert direct neurotoxic effects on primary DRG neurons. However, little is known about paclitaxel’s effects on non-neuronal DRG cells. They provide mechanical and metabolic support and influence neuronal signaling. In the present study, paclitaxel effects on primary DRG non-neuronal cells were analyzed and their concentration or/and time dependence investigated. DRGs of Wister rats (6–8 weeks old) were isolated, and non-neuronal cell populations were separated by the density gradient centrifugation method. Different concentrations of Paclitaxel (0.01 µM–10 µM) were tested on cell viability by MTT assay, cell death by lactate dehydrogenase (LDH) assay, and propidium iodide (PI) assay, as well as cell proliferation by Bromodeoxyuridine (BrdU) assay at 24 h, 48 h, and 72 h post-treatment. Furthermore, phenotypic effects have been investigated by using immunofluorescence techniques. Paclitaxel exhibited several toxicological effects on non-neuronal cells, including a reduction in cell viability, an increase in cell death, and an inhibition of cell proliferation. These effects were concentration- and time-dependent. Cellular and nuclear changes such as shrinkage, swelling of cell bodies, nuclear condensation, chromatin fragmentation, retraction, and a loss in processes were observed. Paclitaxel showed adverse effects on primary DRG non-neuronal cells, which might have adverse functional consequences on sensory neurons of the DRG, asking for consideration in the management of peripheral neuropathy.

## 1. Introduction

Many chemotherapeutic agents may trigger chemotherapy-induced peripheral neuropathy (CIPN), which manifests as tingling, numbness, and burning pain in both hands and feet [1]. The high incidence of CIPN [2] frequently results in dose reduction or the discontinuation of chemotherapy regimens [2,3,4]. Additionally, CIPN symptoms can continue for a very long time after chemotherapy, significantly lowering patients’ quality of life [5].

Sensory neurons are more vulnerable to the toxic effects of anticancer drugs, and patients with CIPN typically experience more sensory symptoms than those in the motor or autonomic systems [6,7]. Chemotherapeutic drugs cause toxicity in myelin sheaths (myelopathy), sensory cell bodies (neuronopathy), and axonal compartments (axonopathy) in the DRG by affecting ion channels, microtubules, mitochondria, and associated capillaries [7,8]. DRG explants have thus been demonstrated to be a good, simple, and well-accepted model for studying peripheral neuropathy caused by antineoplastic agents [9,10,11]. Peripheral sensory (somatic) neurons can easily be reached by chemotherapy drugs as they are located outside the central nervous system without a brain–blood barrier and show strong vascularization due to fenestrated capillaries [12]. Additionally, chemotherapeutic drugs accumulate more in the sensory ganglia than in the peripheral nerves [13,14]. DRG explants’ ability to outgrow neurites in vitro, as well as their response to toxic substances with neurite shortening, make them a reliable model in drug neurotoxicity assessment [9,15,16,17].

Primary DRG cultures consist of a diverse population of cells, including differentiated sensory post-mitotic neuronal cells (neurons) and proliferative non-neuronal cells (Satellite glial cells (SGCs), Schwann cells (SCs), and other glial cells) [18,19,20,21]. In parallel to the valuable impact of neurons, DRG non-neuronal cells are increasingly recognized as important in the development and maintenance of neuropathic pain [22,23]. SGCs, for instance, provide mechanical and metabolic support for neurons by forming an envelope surrounding their cell bodies [14,24]. Therefore, they closely monitor neuronal functions and interact with neurons using both diffusible (e.g., the paracrine release of glial modulators) and non-diffusive mechanisms (e.g., gap junctions) [25,26,27,28]. After nerve injury, SGCs become activated and contribute to the development of neuropathic pain [22,29]. SCs aid in myelinating axons, eliminate cellular debris [30], and play an important role in the outgrowth and guidance of re-growing peripheral axons [31]. SCs not only physically support the long axons, but they also have several growth factors that nourish and myelinate the large associated axons [32,33,34].

Paclitaxel is one of a wide range of commonly used chemotherapeutic agents. Although it has anti-tumoral properties, it also has significant adverse effects on the peripheral nervous system, causing peripheral neuropathy [2,17,35,36]. Paclitaxel shows neurotoxic effects on DRG neurons, including a significant reduction in neurite length and an increase in neuronal cell bodies at different investigated time points, as reported earlier [17,37]. The effects of paclitaxel on neuronal survival and neurite length in the DRG are shown to be dose- and time-dependent [17,37,38]. However, little is known about the effects of paclitaxel on primary DRG non-neuronal cells. The question is still open as to whether similar paclitaxel toxicity in primary DRG non-neuronal cells exists.

Previous research measured the process areas of non-neuronal cells of the DRG inside the mixed culture of neuronal and non-neuronal cells after 24 h of exposure to paclitaxel and found a decrease in the process areas of the non-neuronal cells [39]. In addition, paclitaxel has been shown to reduce cell viability and change the phenotype of SCs isolated from the sciatic nerve at 24 h and 48 h [31]. A recent study also investigated the impact of paclitaxel on the viability and proliferation of SGCs and found no effect on viability but a decrease in cell proliferation [14]. However, more research is needed to fully understand paclitaxel toxicity in the entire culture of non-neuronal cells (SCs, SGCs, and other glial cells). These outcomes may shed more light on the potential functional consequences of paclitaxel on primary DRG sensory neurons and the therapeutic interventions for peripheral neuropathy.

Therefore, the aim of this study was to investigate the effects of paclitaxel on primary DRG non-neuronal cells and determine the time course of those changes. DRG non-neuronal cells were isolated and treated with different concentrations of paclitaxel at different time points. Effects on viability, morphology, and proliferation were analyzed. We applied approaches such as the MTT assay to study cell viability [40], the lactate dehydrogenase (LDH) assay [41], and the propidium iodide (PI) assay to study cell death [42], as well as Bromodeoxyuridine (BrdU), to study cell proliferation [43]. These approaches are frequently employed in related studies [14,44,45,46]. We hypothesized that paclitaxel exposure would have severe toxic effects on DRG non-neuronal cells, which might be dose- or/and time-dependent.

## 2. Materials and Methods

### 2.1. Ethics Statement

All research involving animal material was carried out in accordance with the ethics policy and the policy on animal use in neuroscience research as outlined in Directive 2010/63/EU of the European Parliament and of the Council of the European Union on the protection of animals used for scientific purposes and was approved by local authorities for laboratory animal care and use (State of Saxony-Anhalt, Germany, permission number: I11M27).

### 2.2. Materials

Paclitaxel was used and administered into culture media in accordance with the treatment protocol (Taxol equivalent, Invitrogen, cat No. P3456-5 mg, Schwerte, Germany). Dimethyl sulfoxide (DMSO, Sigma–Aldrich, cat. No. D4540-500 mL, Lyon, France) was used to dissolve paclitaxel to obtain stock solutions of 1 mM and stored at −20 °C.

### 2.3. Isolation and Preparation of Primary DRG Co-Culture

DRG tissues were isolated from Wister rats aged 6–8 weeks. Rats were deeply anesthetized before scarification by isoflurane (Florene, 100% (*v/v*), 250 mL, Abcam, cat No. B506, France). Under aseptic conditions, the vertebral column was isolated, and all surrounding muscle, fat, and soft tissue were carefully removed. The spinal cord was exposed, and after that, DRGs were located, removed, and collected from intervertebral foramina on both sides in a sterile dish containing 3 mL of Hanks balanced salt solutions without Mg^2+^/Ca^2+^ (HBSS, Invitrogen, REF. 24020-091, Schwerte, Germany) (Figure S1). The culture of non-neuronal cells was conducted in accordance with a previously published protocol [47], with some modifications. In brief, isolated DRGs were enzymatically digested in the first enzymatic solution containing 60 U/mL papain (Sigma–Aldrich, cat No. P4762-100 mg, St. Louis, MO, USA), 3 µL of 80 mg/mL saturated sodium hydrogen carbonate solution (NaHCO_3_, Merck, cat No. k22399729, Darmstadt, Germany), and 0.6 mg/mL L-Cysteine (L-Cys, Sigma–Aldrich, Cat No. C7352-25 g, St. Louis, MO, USA) dissolved in 1.5 mL of HBSS without Mg^2+^/Ca^2+^. DRGs were then incubated for 15 min in a 37 °C water bath before being incubated in the second solution containing 4 mg/mL collagenase type II solution (CLS2, Sigma–Aldrich, Cat No. C6885-1 gm, St. Louis, MO, USA) and 4.6 mg/mL dispase type II (Dispase II, Sigma–Aldrich, Cat No. D4693-1 gm, St. Louis, MO, USA) solution in 3 mL HBSS without Mg^2+^/Ca^2+^. The DRGs were gently mixed with collagenase solution and incubated for an additional 15 min at 37 °C.

The resulting cell suspension underwent a one-minute centrifugation at 200 g. After carefully aspirating the collagenase solution, the DRGs were triturated 10–15 times with 1 mL of F12 medium (1X, Invitrogen, REF.11765-054, Schwerte, Germany) supplemented with 10 % of heat-inactivated Fetal Bovine Serum (FBS, Invitrogen, REF. 10270-106, Schwerte, Germany) and 1 % of 0.1 mg/mL streptomycin/penicillin (Sigma–Aldrich, cat No. P4333/100 mL, Darmstadt, Germany) by using 1000 µL pipette tips till the cell suspension became cloudy.

### 2.4. Seeding and Growth of Primary DRG Co-Culture

**Figure 1:**
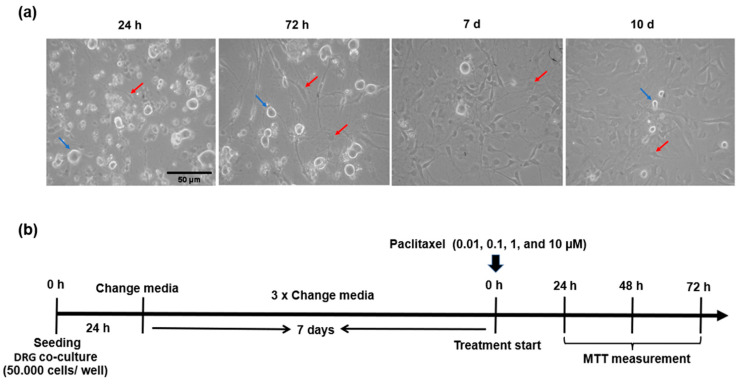
Morphological features and treatment protocol of primary DRG co-culture. (**a**) Representative images show the morphology and growth of DRG co-culture at different time points, blue arrows indicate neuronal populations, while red arrows indicate different subpopulations of DRG non-neuronal cells, Scale bar = 50 µm. (**b**) Treatment protocol for studying the effects of paclitaxel on DRG co-culture viability by using MTT assays at 24 h, 48 h, and 72 h post-treatment.

Circular coverslips were pre-coated for at least 1 h or overnight at 4 °C with 2 mg/mL Poly-D-lysine (PDL, Sigma–Aldrich, cat No. P6407, St. Louis, MO, USA) and 0.2 mg/mL laminin (Sigma–Aldrich, cat No. L2020-1 mg, St. Louis, MO, USA), then washed once with dist. H_2_O and added directly before seeding cells in the culture medium. DRGs (50,000 cells) co-cultured in 50 µL culture medium were then pre-seeded on the coated coverslips for 2 h in an incubator at 37 °C and with 5% CO_2_. One mL of warm culture medium adjusted to pH 7.4 was gently added to cells per well and maintained at 37 °C with 5% CO_2_. Growth and morphology of co-cultivation of neurons and non-neurons were observed after 24 h, 72 h, 7 days, and 10 days (Figure 1a).

### 2.5. Effects on Cell Viability of Primary DRG Co-Culture (MTT Assay)

DRG co-cultured cells (5 × 10^4^ cells/well) were treated 8 days after seeding with different concentrations of paclitaxel (0.01–10 µM) at 24 h, 48 h, and 72 h post-treatment in 96 well plates to study the effects on cell viability (Figure 1b). Four concentrations were then chosen that were as close to clinically applied doses as possible. Furthermore, the selected paclitaxel concentrations are in line with earlier reports from the literature [37,38,39,48,49,50,51]. Cell viability (%) was measured at 24 h, 48 h, and 72 h post-treatment using MTT assay. Four hours prior to the end of the experiments at various time points, 3-(4,5-dimethylthiazol-2-yl)-2,5-diphenyltetrazolium bromide solution (MTT, Invitrogen, cat. No M6494, 5 mg/mL, Eugene, OR, USA) was added. After an additional 4 h of incubation, the MTT solution was removed from the cells, and formazan crystals were dissolved in 100 µL of DMSO. Absorbance values were determined at two wavelengths (540 nm and 720 nm) by a microplate reader (SynergyTMMx, BioTek Instruments, Winooski, VT, USA) after another 20 min. Co-cultures maintained in standard media without paclitaxel were used as the control group. To rule out any effects of the solvent on cell viability, controls had DMSO at the same highest concentration (0.1%) as those used in other groups. For each treatment, three technical replicas were used in three biologically independent experiments.

### 2.6. Separation of Primary DRG Non-Neuronal Cells

To separate non-neuronal cells, density gradient centrifugation was applied by using bovine serum albumin (BSA, Sigma Aldrich, cat No.A7906-10 G, St. Louis, MO, USA) (15% (*w/v*) BSA solution) for purification [52]. The DRGs were triturated 10–15 times in 1 mL of high-glucose Dulbecco’s Modified Eagle Medium (DMEM; Invitrogen; Ref. 41965-039; Schwerte, Germany) supplemented with 10 % FBS. Non-neuronal cells were separated from the DRG mixed culture by centrifuging single-cell suspensions through a 15% (*w/v*) BSA solution in DMEM. One milliliter of cell suspension was added to three milliliters of 15% BSA solution in a 15 mL conical tube and centrifuged at 300 g for 8 min at room temperature (RT) (Figure S1b). Thereafter, the layer of non-neuronal cells was carefully transferred to a 15 mL conical tube by using 1000 µL pipette tips. Then, 1 mL of warmed F12 medium supplemented with 10% FBS and 1% of 0.1 mg/mL streptomycin/penicillin was added, and the DRG non-neurons were suspended. A 40 µm cell strainer (SARSTEDT, cat. no. D-51588, Schwerte, Germany) was then used to filter the cell suspension to remove cell debris and undigested tissue fragments.

### 2.7. Seeding and Growth of Primary DRG Non-Neuronal Cells

Sterilized 12 mm circular coverslips were used, and they were washed and dried once with dist. H_2_O. 50,000 cells resuspended in 50 µL culture medium were then pre-seeded on the sterilized coverslips for 2 h in an incubator at 37 °C and with 5% CO_2_. One mL of warm culture medium adjusted to pH 7.4 was gently added to the cells, which were then preserved at 37 °C with 5% CO_2_. Growth and morphology of DRG non-neuronal cells were observed after 24 h, 48 h, 72 h, 96 h, and 7, 10 days.

### 2.8. Effects of Paclitaxel on DRG Non-Neuronal Cells

#### 2.8.1. Cell Viability (MTT Assay)

**Figure 2:**
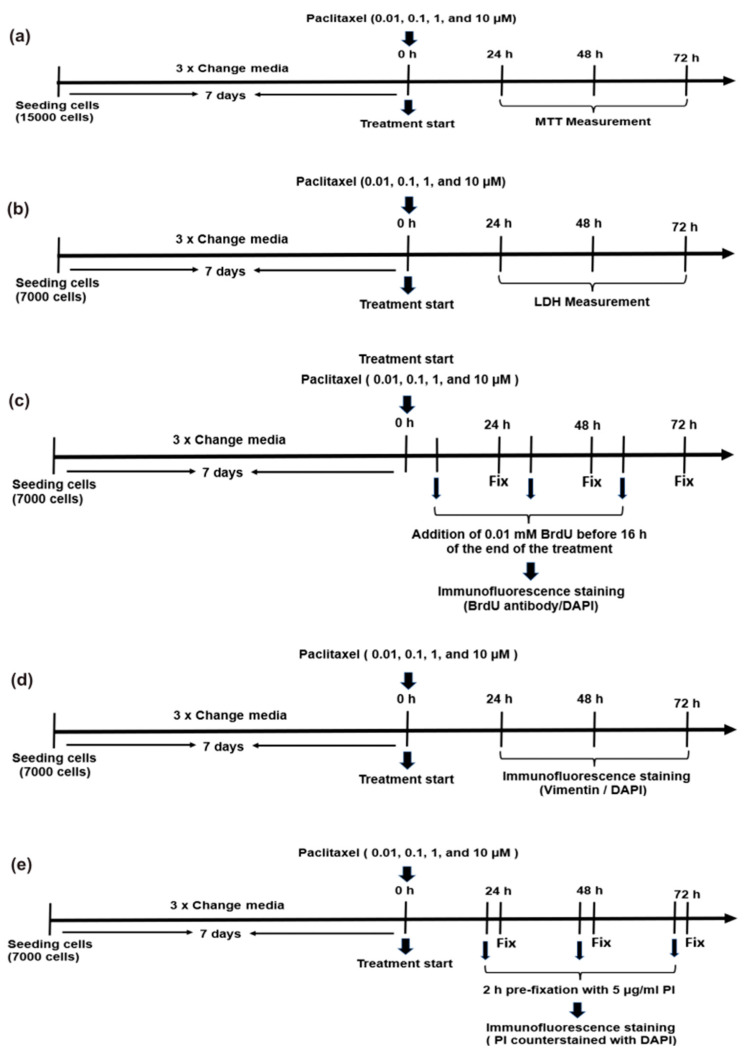
Various treatment protocols investigate the effects of different paclitaxel concentrations on primary DRG non-neuronal cells after 24 h, 48 h, and 72 h of the application. (**a**) The MTT assay was used for cell viability determination; (**b**) the LDH assay for cytotoxicity measurements; (**c**) the BrdU assay was used to detect cell proliferation; (**d**) treatment protocol for studying the effects of paclitaxel on cellular morphology through immunofluorescence staining; (**e**) detection of cell death by using the PI assay.

In 96 well plates, non-neuronal cells (15 × 10^3^ cells/well) were seeded for 7 days, followed by treatment with four different concentrations of paclitaxel (0.01 µM, 0.1 µM, 1 µM, and 10 µM) at three different time points: 24 h, 48 h, and 72 h post treatment (Figure 2a). The effects of paclitaxel on the cell viability of non-neuronal cells were measured by MTT assay, as described above in Section 2.5.

#### 2.8.2. Determination of Cytotoxicity (LDH Assay)

In 24 well plates, DRG non-neuronal cells (7 × 10^3^ cells/well) were seeded in DMEM/F12 free phenol red medium (1X, Gibco, REF.21041-025, Schwerte, Germany) supplemented with 10 % inactivated FBS and 1% of 0.1 mg/mL streptomycin/penicillin for 7 days, followed by treatment with four different concentrations of paclitaxel (0.01 µM, 0.1 µM, 1 µM, and 10 µM) prepared in culture media supplemented with 1% FBS at different time points: 24 h, 48 h, and 72 h post treatment (Figure 2b). Additional wells were filled without cells for culture media control (blank). For determination of maximum LDH release (positive LDH control, 100 % cell death), 1:10 of the LDH lysis kit (LDH, Sigma Aldrich, cat. No. TOX7, St. Louis, MO, USA) was added to some wells and incubated for 45 min. According to the manufacturer’s instructions, culture media samples from cells or controls at certain time points were transferred to 1.5 mL tubes and then centrifuged at 250× *g* for 4 min to pellet cells. Afterward, 40 µL of the supernatant of different samples was added in 5 replicates to a clean flat-bottom 96-well plate and proceeded with enzymatic analysis. The LDH assay mixture was prepared at the time of use by adding 20 µL per well. The plates were covered with aluminum foil for light protection and incubated at room temperature for 30 min. The reaction was then stopped by adding 6 µL of 1 N Hydrochloric acid (HCl, Sigma Aldrich, cat. No. H9892, St. Louis, MO, USA) to each well. Absorbance values of samples were measured at a wavelength of 490 nm and the background absorbance of multi-well plates at 690 nm. Background absorbance values were subtracted from the primary wavelength measurements (490 nm). Finally, all controls, samples, and maximal measurements were normalized with blank measurements. Then the percent of cytotoxicity was calculated according to the below equation [53].

$$\%\;Cell\;death=\frac{\left(sample\;absorbance\;value-mean\;control\;value\right)}{\left(mean\;complete\;kill\;result-mean\;control\;value\right)}\;\times \;100$$

#### 2.8.3. Detection of Cell Proliferation by BrdU Assay

To investigate the effects of paclitaxel on cell proliferation, DRG non-neuronal cells (7 × 10^3^ cells/well) were seeded on 12 mm sterile coverslips in a 24 well plate, cultured for 7 days, and treated with various concentrations of paclitaxel at different time windows. Four µL of 0.01 mM 5-bromo-2′-deoxyuridine (BrdU, Sigma Aldrich, cat No. B5002-1G, St. Louis, MO, USA) was added to each well 16 h before fixation (Figure 2c). Cells were either immediately subjected to immunofluorescence or stored in 0.02 M PBS at 4 °C pending further use after fixation with 4% paraformaldehyde (PFA, AppliChem, cat No. 141451.1211, Darmstadt, Germany) for 15 min at room temperature. For labeling, non-specific bindings were blocked with normal goat serum (NGS, Sigma–Aldrich, cat. No. G9023-10 mL, Taufkirchen, Germany, 1:20) in 0.02 M PBS/0.3% (*v/v*) plus triton X-100 for 30 min. Thereafter, cells were washed three times with 0.02 M PBS for ten minutes each and incubated with a monoclonal mouse anti-BrdU antibody (Dako, cat. No. M0744-1 mL, Glostrup, Denmark, 1:200) overnight. Coverslips were then incubated with the goat anti-mouse Alexa Fluor^®^ 488 conjugated secondary antibody (Life Technologies, cat. no. 2066710, Darmstadt, Germany, 1:200) for 1 h washed three times with PBS/triton for ten minutes. By using DAPI (4′,6-Diamin-2-phenylindol, Sigma–Aldrich, Munich, Germany, cat No. D9542), nuclei were visualized, and coverslips were mounted with DAKO fluorescence mounting medium (DAKO, Agilent Technologies, Inc., Santa Clara, CA 95051, USA). A confocal laser scanning microscope (Leica DMi8, Wetzlar, Germany) was used to take photomicrographs from five to eight randomly chosen areas. BrdU-positive cells were manually counted with Image J’s multipoint tool (version 1.46r, National Institutes of Health, Laboratory for Optical and Computational Instrumentation, University of Wisconsin, Madison, WI, USA), and the percentage of proliferating cells was determined by dividing the number of BrdU^+^ cells by the total number of DAPI-stained nuclei. To obtain the data, three independent experiments were conducted.

#### 2.8.4. Determination of Paclitaxel Effects on Cellular Morphology

To study the effects of paclitaxel on the morphology of DRG non-neuronal cells, cells (7 × 10^3^ cells/well) were seeded on 12 mm sterile coverslips in a 24 well plate, cultured for 7 days to allow nearly all cells to proliferate, and then treated with various concentrations of paclitaxel at different time windows (Figure 2d). After fixation, the immunofluorescence staining procedure was followed as described in Section 2.8.3. Chicken anti-vimentin polyclonal primary antibody (Abcam, cat No. ab24525, Cambridge, UK, 1:1000) combined with goat anti-chicken IgY Alexa Fluor^®^ 488 conjugated (Invitrogen, REF. A11039-0.5 mL, Eugene, OR, USA, 1:200) as secondary antibody was used for labeling the cytoskeleton of non-neuronal cells. Then the procedure is completed as previously described in Section 2.8.3. Images were taken with a Leica confocal laser scanning microscope (Leica DMi8, Wetzlar, Germany), and five to eight areas were randomly captured per coverslip in three independent experiments.

#### 2.8.5. Analysis of Apoptosis by Assessment of Nuclear Morphology

DRG non-neuronal cells were stained with the DNA dye DAPI to visualize nuclear morphology. The percentage of apoptosis (early and late apoptosis) was quantitated by scoring the percentage of apoptotic cells in the adherent cell population. Stained nuclei with a uniform and regular morphology were scored manually as healthy and viable cells. Cells with condensed, fragmented, or blubber nuclei were scored as apoptotic cells. The total number of nuclei in non-neuronal cells was counted automatically using Fiji software (https://imagej.net/Fiji/Downloads). After converting DAPI images into 8-bit gray scale images, the threshold of nuclei was adjusted manually, and the separation of attached nuclei was performed by applying a binary watershed. Finally, the analyzing particles option was applied, and the total number of nuclei was determined per image (Figure S2). Photomicrographs were captured using a Leica (DMi8, Wetzlar, Germany) confocal laser scanning microscope, and five to eight areas were recorded per each coverslip randomly in three independent experiments.

#### 2.8.6. Detection of Cell Death by Propidium Iodide Staining

For detection of degenerating non-neuronal nuclei of dead cells by late apoptosis or necrosis, cells (7 × 10^3^ cells/well) were seeded on 12 mm sterile coverslips in a 24 well plate, cultured for 7 days, and then treated with various concentrations of paclitaxel at 24 h, 48 h, and 72 h after treatment. Then, 5 µg/mL propidium iodide (PI, Merk, cat No. 537059-50 mg, Darmstadt, Germany) was added 2 h before fixation. Afterwards, cells were washed three times with PBS and then fixed with 4% PFA for 15 min (Figure 2e). Coverslips were washed three times with PBS/triton and incubated with DAPI. All stained slides were washed with aqua distilled water before being covered with a DAKO fluorescence mounting medium. Images were captured using a Leica (DMi8, Wetzlar, Germany) confocal laser scanning microscope, and five to eight areas were recorded per each coverslip randomly in three independent experiments. For the detection of PI-labeled dead cells, monochromatic light at 543 nm and an emission bandpass filter of 585–615 nm was used. PI-positive cells were counted manually using the multipoint tool of Image J software version v1.46r.

### 2.9. Statistical Analysis

GraphPad Prism 8.0.1 for Windows (GraphPad Software, La Jolla, CA, USA, www.graphpad.com, accessed on 22 May 2023) was used for data analysis and visualization. All the data were checked for normality using the Kolmogorov–Smirnov test. Statistics were performed using a one-way ANOVA (analysis of variance) followed by a Bonferroni post-test, with significance set at *p* < 0.05. All tests had an alpha level of 0.05.

## 3. Results

### 3.1. Characterization of Primary DRG Co-Culture

The growth of DRG co-culture was checked at different timelines (1, 3, 7, and 10 days) by a light microscope. DRG co-culture is a heterogeneous population of neuronal and non-neuronal cells. DRG neurons were characterized by refractile and bright cell bodies, and three different subpopulations were observed according to the size of their somata (small, ≤599 µm^2^; medium, 600–1199 µm^2^ and large, 1200–1300 μm^2^), which represented 67%, 31%, and 2% of neurons in culture, respectively [37]. Additionally, three different subpopulations of DRG non-neuronal cells were observed in the culture (SCs, SGCs, and fibroblasts) (Figure 1a).

### 3.2. Effects of Paclitaxel on Viability of Primary DRG Co-Culture by MTT Assay

**Figure 3:**
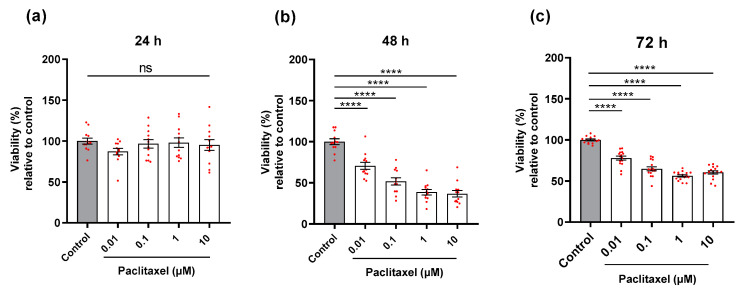
Effects of different paclitaxel concentrations on viability (%) of DRG co-culture at 24 h, 48 h, and 72 h post-treatment by MTT assay. (**a**) No significant effect on viability was found in co-cultures compared to controls at 24 h post-treatment (*p* > 0.05). (**b**) 48 h, and (**c**) 72 h post-treatment, paclitaxel displayed a significant reduction in the viability of cells compared to the control (**** *p* < 0.0001). The asterisks depict statistically significant results regarding the respective measurement indicated with the bar. Values are served as the mean ± SEM of three independent experiments performed in triplicate. ns, non-significant.

DRG co-cultures (neurons and non-neuronal cells) were treated with different concentrations of paclitaxel for 24 h, 48 h, and 72 h post-treatment. At 24 h post-treatment, the four different concentrations of paclitaxel showed no significant effects on the viability of DRG co-culture in comparison with the control group (*p* > 0.05) (Figure 3a). However, all paclitaxel concentrations demonstrated a significant suppression in the viability of cells in DRG co-culture compared to the control group at 48 h and 72 h post-treatment (*p* < 0.0001) (Figure 3b,c).

### 3.3. Characterization of Primary DRG Non-Neuronal Cells

**Figure 4:**
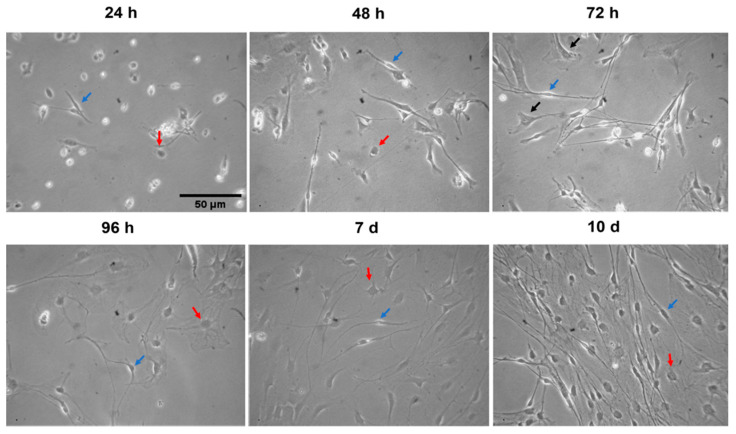
Representative phase contrast images show the morphology and growth of primary DRG non-neuronal cells at various time points. Blue arrows indicate Schwann cells, red arrows satellite glial cells, and black arrows represent fibroblasts, Scale bar = 50 µm.

DRG non-neuronal cells were examined under a light microscope at different time points (1, 2, 3, 4, 7, and 10 days) to analyze their growth and morphology. DRG non-neuronal cells are divided into three different subpopulations. The first population are SCs, which represent the majority of DRG non-neuronal cells [20,21]. They are distinguished by a single, small, spindle-shaped nucleus. These cells have a thin layer of cytoplasm surrounding the nucleus and bipolar cell bodies with long, thin projections or processes extending from each side. These long processes can either form a dense bundle of fibers or travel in a single thread of fibers away from the cell body (Figure 4). The population of SGCs shows small, round, and flat cell bodies with wide cytoplasmic projections ***(***Figure 4). These cells play a crucial role in the formation of an enveloping layer around DRG neurons for protection and metabolism. Lastly, fibroblasts are found under SCs with a large flat cell body and are pyramidal in shape with multipolar wide projections that are not associated with any other fibers. These cells are secretory active and form the connective tissue that supports cells in the culture (Figure 4).

### 3.4. Effects of Paclitaxel on Primary DRG Non-Neuronal Cells

#### 3.4.1. Cell Viability (MTT Assay)

**Figure 5:**
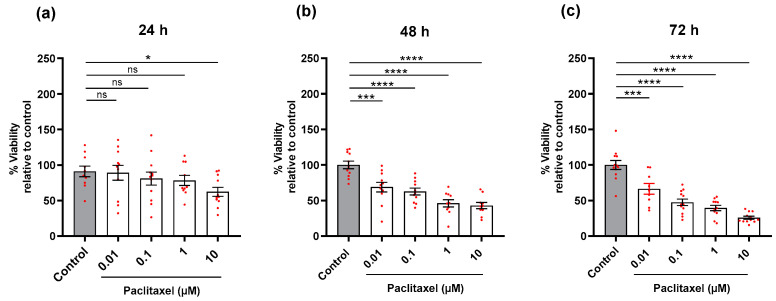
Effects of different concentrations of paclitaxel on the viability (%) of DRG non-neuronal cultures at 24 h, 48 h, and 72 h post-treatment by using MTT assay. (**a**) 10 µM of paclitaxel was the only concentration that showed a significant effect on the viability of DRG non-neuronal cells compared to control at 24 h post-treatment (* *p* < 0.05). (**b**,**c**), Different concentrations of paclitaxel elucidated a significant reduction in the viability of cells compared to the control at 48 h and 72 h post-treatment (*** *p* < 0.001, **** *p* < 0.0001). The asterisk denotes significant results regarding the respective measurement indicated with the bar. Values are served as mean ± SEM of three independent experiments performed in triplicate, ns: non-significant.

DRG non-neuronal cells were exposed to various concentrations of paclitaxel for 24 h, 48 h, and 72 h post-treatment. Only 10 µM of paclitaxel showed a significant reduction in the viability of cells compared to the control group at 24 h post-treatment (*p* < 0.05) (Figure 5a). While, at 48 h and 72 h post-treatment, different paclitaxel concentrations showed a significant reduction in the viability of non-neuronal cells compared to the untreated control group (*p* < 0.05) (Figure 5b,c). At 72 h post-treatment, the effects of paclitaxel on the viability of non-neuronal cells were clearly concentration-dependent (Figure S3a). Notably, the effects of 10 µM paclitaxel on the viability of non-neuronal cells were only time- but not concentration-dependent (Figure S3b).

#### 3.4.2. Determination of Cytotoxicity (LDH Assay)

**Figure 6:**
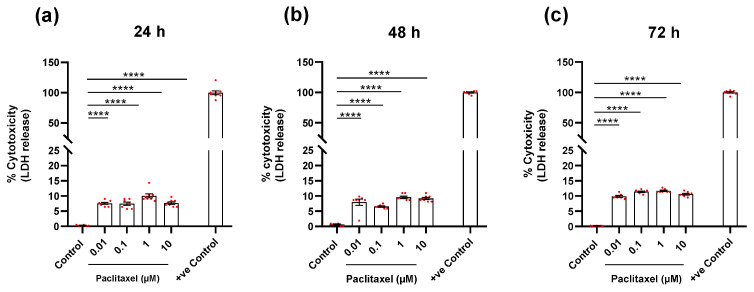
Effects of different concentrations of paclitaxel on cytotoxicity of DRG non-neuronal cultures using lactate dehydrogenase (LDH) assay. Levels of released LDH were quantified at (**a**) 24 h, (**b**) 48 h, and (**c**) 72 h post-treatment and showed a significant increase in LDH release that was proportional to the number of dead or damaged cells compared to the control group (**** *p* < 0.0001). +ve Control represents the maximum release of LDH after 100% cell death. The asterisks denote significant results regarding the respective measurement indicated with the bar. Values are given as the mean ± SEM of three independent experiments conducted in 15 replicates.

The treatment of DRG non-neuronal cells with different paclitaxel concentrations (0.01 µM, 0.1 µM, 1 µM, and 10 µM) resulted in a significant increase in the number of damaged or dead cells that was proportional to the amount of LDH released in the cell culture media compared to non-treated cells (*p* < 0.0001) at 24 h after treatment (Figure 6a). After 48 h of treatment, the cytotoxicity of the four concentrations of paclitaxel increased remarkably compared to the control (*p* < 0.0001) (Figure 6b). The increase in the number of dead cells in response to the exposure of non-neuronal cells to paclitaxel continued in comparison to the control group (*p* < 0.0001) at 72 h post-treatment (Figure 6c). It was obvious that the effects of different paclitaxel concentrations on cytotoxicity were dose-dependent at only 72 h post-treatment (Figure S4a). Furthermore, a considerable difference was observed between different investigated time points for all applied concentrations, indicating time-dependent effects (Figure S4b).

#### 3.4.3. Cell Proliferation by BrdU Assay

**Figure 7:**
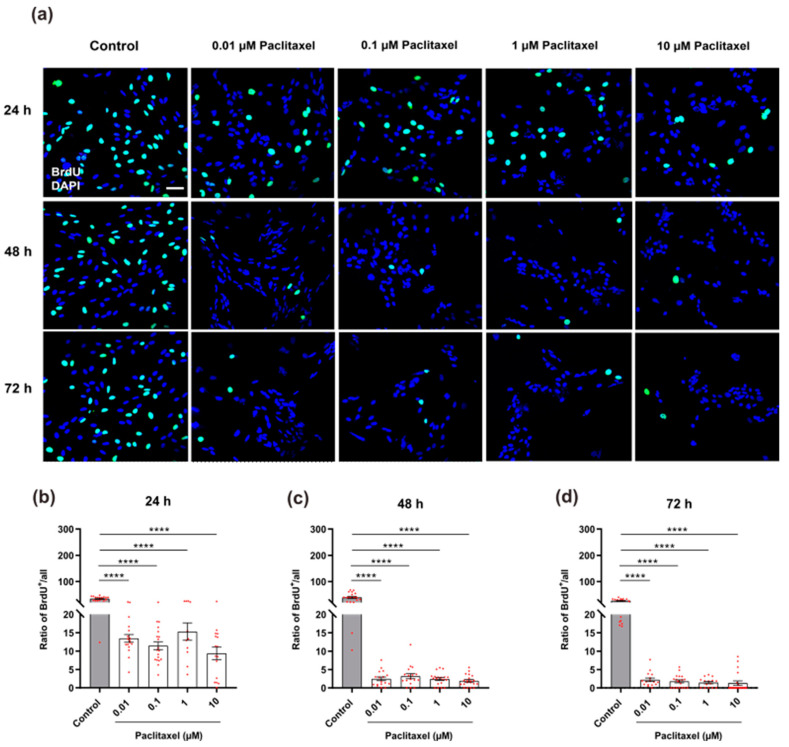
Effects of different concentrations of paclitaxel on cell proliferation of DRG non-neuronal cells using BrdU assay. (**a**) Representative immunofluorescence images of different non-neuronal cells treated with 0.01 µM, 0.1 µM, 1 µM, and 10 µM paclitaxel at 24 h, 48 h, and 72 h post-treatment show proliferating cells labeled with BrdU antibody (green) and all nuclei stained with DAPI (blue). 5–8 areas were recorded randomly per each coverslip; Scale bar = 75 µm. Bar charts demonstrated a significant decrease in the rate of cell proliferation after treatment compared to the control group (**** *p* < 0.0001) at (**b**) 24 h, (**c**) 48 h, and (**d**) 72 h post-treatment. The asterisks denote significant results regarding the respective measurement indicated with the bar. Values served as the mean ± SEM of three independent experiments performed in 15 replicates.

The percentage of BrdU immunoreactive cells was determined in non-neuronal cells after exposure to various concentrations of paclitaxel at 24 h, 48 h, and 72 h post-treatment. At all investigated time points, a significantly lower number of BrdU-positive cells was found in treated cultures with different paclitaxel concentrations compared to the vehicle control group (*p* < 0.0001) (Figure 7a–d). As no significant difference was detected between different paclitaxel concentrations, no concentration-dependent effect was assumed (Figure S5a). In contrast, a significant difference between different timelines for all applied concentrations of paclitaxel was found, revealing a time-dependency of anti-proliferative effects (Figure S5b).

#### 3.4.4. Cellular Morphological Changes

**Figure 8:**
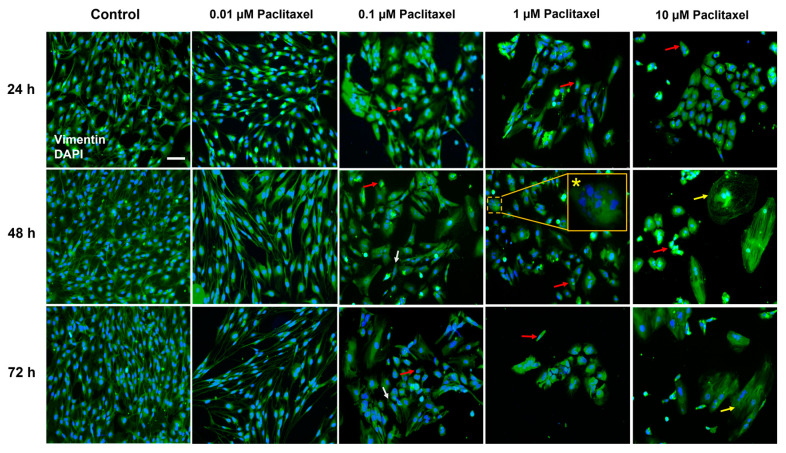
Effects of various paclitaxel concentrations on cellular morphology at different investigated time points using immunofluorescence staining. Representative microphotographs demonstrate cells stained with vimentin antibody (green) and nuclei counterstained with DAPI (blue). Paclitaxel (0.1 µM, 1 µM, and 10 µM) strongly affected the cell morphology of non-neuronal cells including shrinkage of cells’ bodies (red arrows) and retraction of processes (white arrows). In addition, some cells treated with 10 µM paclitaxel were swelling (yellow arrows). Additionally, nuclear changes were observed, such as nuclear fragmentation (indicated by an asterisk in the inlet) and condensation. Five to eight regions were recorded randomly per coverslip by fluorescence microscopy. Scale bar = 75 µm.

Except for 0.01 µM, all applied paclitaxel concentrations showed hallmarks of cell death and a variety of toxic alterations to the morphology of non-neuronal cells, including cell shrinkage, swollen cell bodies, or reductions in the length of processes. Additionally, other morphologic changes were observed in nuclei, such as nuclear fragmentation and chromatin condensation (Figure 8). The number of viable DRG non-neuronal cells was significantly reduced (*p* < 0.05) compared to the control group at all time windows (Figure 8).

#### 3.4.5. Analysis of Changes in Nuclear Morphology

**Figure 9:**
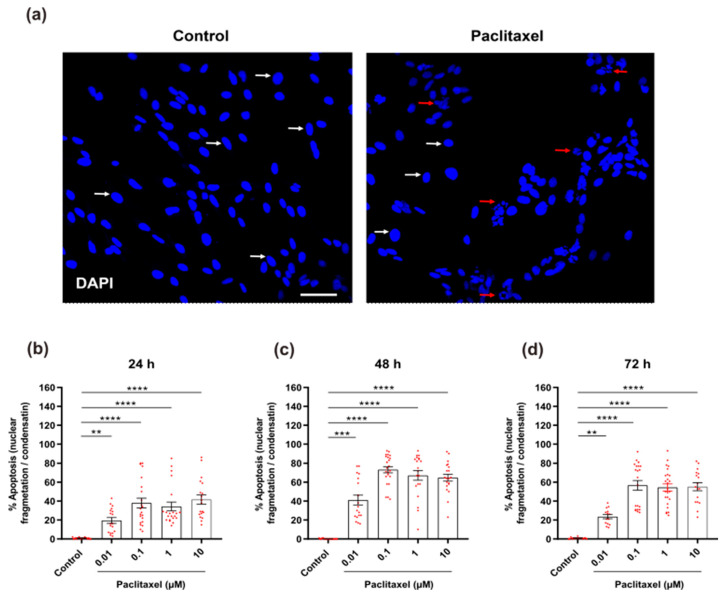
Effects of different concentrations of paclitaxel on nuclear morphology of DRG non-neuronal cells were analyzed by DAPI staining to detect % of apoptosis. (**a**) Representative images show DAPI-stained nuclei of non-neuronal cells of the control group (left) or 1 µM paclitaxel group (right) at 48 h post-treatment, Scale bar = 75 µm. White arrows indicate healthy and uniformly stained nuclei, whereas red arrows identify apoptotic nuclei. (**b**–**d**) A significant increase in % of apoptotic cells with fragmented or condensed nuclei was observed in different cultures treated with various paclitaxel concentrations (0.01 µM, 0.1 µM, 1 µM, and 10 µM) in comparison with the control group (** *p* < 0.01, *** *p* < 0.001, **** *p* < 0.0001). Data represented as mean ± SEM. The experiments were performed at least three independent times with n = 15 replicas. The asterisk denotes significant results regarding the respective measurement indicated with the bar graphs.

The effects of paclitaxel on nuclear morphology were investigated 24 h, 48 h, and 72 h after treatment. Paclitaxel induces nuclear fragmentation and condensation, which are hallmarks of apoptosis (Figure 9a). Different paclitaxel concentrations revealed a substantially increased number of apoptotic cells when compared to the control group (*p* < 0.05) at all investigated time points (Figure 9b–d). There was a significant difference between paclitaxel concentrations, indicating concentration dependence at the various time points studied (Figure S6a). Moreover, there was a significant difference between different investigated time windows, particularly between 24 h and 48 h for all paclitaxel concentrations, indicating a time dependence for the effects of different paclitaxel concentrations (Figure S6b).

#### 3.4.6. Detection of Cell Death by Propidium Iodide Staining

**Figure 10:**
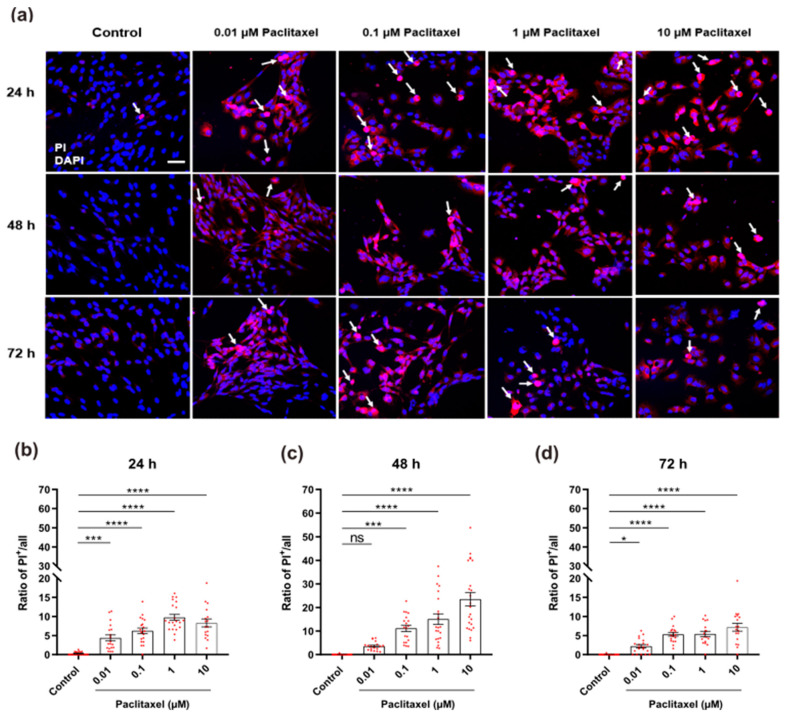
Effects of different paclitaxel concentrations on cell death of DRG non-neuronal cells by using PI assay. (**a**) Representative immunofluorescent fields show the amount of damaged non-neuronal cells (PI-positive) in treated groups compared to control fields. The white arrows represent degenerating cells (bright pink nuclei), Scale bar = 75 µm. At 24 h (**b**)**,** 48 h (**c**), and 72 h (**d**) post-treatment, all concentrations of paclitaxel led to a massive increase in the number of dead cells compared to the control group (* *p* < 0.05, *** *p* < 0.001, **** *p* < 0.0001), except for 0.01 µM paclitaxel concentration at 48 h (*p* > 0.05). Values served as mean ± SEM, and the experiments were carried out three times independently with n = 15 replicas. The asterisk denotes significant results regarding the respective measurement indicated with the bar graphs, ns: non-significant.

To detect degenerating non-neuronal cells with late apoptosis and necrosis, combined staining with PI and DAPI was performed. Dead cells showed pycnotic highly condensed or fragmented nuclei in bright pink, while live cells showed normal nuclei with homogenously distributed chromatin and regular morphology (Figure 10a). Except for 0.01 µM at 48 h post-treatment, all treated groups at all time points showed an apparent increase in the ratio of positive PI cells when compared to their corresponding untreated control group (*p* < 0.05) (Figure 10b–d). The presence of dead cells also increased with increasing paclitaxel concentrations when compared to the control, confirming a concentration dependency at different investigated time points (Figure S7a). Furthermore, a time-dependent increase in the ratio of cell death to DRG non-neuronal cells was observed except for 0.01 µM paclitaxel at 48 h (Figure S7a).

## 4. Discussion

Primary DRG non-neuronal cells play a crucial role in supporting DRG neurons [22,31]. Previous studies investigated the toxic effects of paclitaxel on Primary DRG neurons, but little is known about the toxicity of paclitaxel on non-neuronal cells. Furthermore, the time course and concentration-dependency of paclitaxel’s toxic effects on non-neuronal cells attracted little attention. To address these aspects, a more comprehensive approach using a variety of techniques, time points, and concentrations was chosen to demonstrate the effects of paclitaxel on DRG non-neuronal cell culture in vitro.

Paclitaxel exhibited several toxicological effects on primary DRG non-neuronal cells, including a decrease in cell viability, an increase in cell death, inhibition of cell proliferation, and cellular and nuclear changes, all of which were concentration- and time-dependent. Our findings on DRG SCs are consistent with previous research that studied paclitaxel effects on viability in a model of isolated SCs from the sciatic nerve [31]. These effects were attributed to paclitaxel’s fast and strong mechanism of action on primary DRG non-neuronal cells, as these cells are non-transformed and proliferating cells. Therefore, paclitaxel selectively induces the death of transformed cells, possibly by arresting the cell cycle at G1 as well as G2/M phases [54,55,56,57,58].

Our results also revealed that paclitaxel significantly reduced the proliferation rate of DRG non-neuronal cells at various investigated timelines regardless of the applied concentration, but this suppression increased in a time-dependent manner. These findings expand the data of previous research, which reported a decrease in cell proliferation of SGCs of DRG after 24 h of treatment with 1 µM and 5 µM paclitaxel [14]. The authors postulated a paclitaxel stabilizing effect on microtubules by binding to beta-tubulin units, which disrupts microtubule dynamics [58]. As a result, mitosis was arrested between metaphase and anaphase (G2/M phase), suggesting a mitotic block and proliferation inhibition [57,58,59].

The majority of anticancer drugs have been shown to induce apoptosis in vulnerable cells [60,61,62]. Cellular and nuclear changes induced by anticancer drugs are very common and involve shrinkage of cell bodies, nuclear condensation, and chromatin fragmentation [54,55,56]. As shown here, the response to paclitaxel seems similar in primary DRG non-neuronal cells and affects all cellular subtypes.

Interestingly, we found that the percentage of apoptotic cells in DRG non-neuronal cell culture detected by DAPI staining at different investigated time points was higher when compared to the proportion of dead cells determined by the PI assay. This seeming discrepancy can be explained as DAPI staining detects cells in the early and late stages of apoptosis based on their nuclear morphology [63], but PI labels late apoptotic and dead cells with damaged cell membranes [64].

Furthermore, retraction and loss or shortening of processes increased strongly with the duration of treatment. These results add to the time- and concentration-dependency of paclitaxel effects and support previous research that reported a loss or shortening of processes in non-neuronal cells, however, in primary DRG co-culture after 24 h of exposure to paclitaxel [31,39]. These effects are comparable to those found in sensory neurons [17,37,65], implying the strong toxicity of paclitaxel on DRG non-neuronal cells, which might have adverse functional consequences for DRG sensory neurons.

Dose- and time-dependent pharmacokinetics have been reported more frequently for anticancer drugs than for other medications [66,67,68,69,70]. Our findings revealed that the effects of paclitaxel on Primary DRG non-neuronal cell culture are concentration- and time-dependent. Previous studies also reported similar findings on primary DRG neuronal and non-neuronal cells [14,17,39]. Low concentrations of paclitaxel (0.01–0.1 µM) were reported to suppress microtubule dynamics and inhibit mitotic spindle formation, resulting in a cell cycle arrest at the G2/M phase [55]. Considerably, low concentrations of paclitaxel showed no effect on the overall architecture of the microtubule cytoskeleton (Jordan et al., 1993), as noticed with 0.01 µM paclitaxel in the current study. In contrast, higher doses of paclitaxel were found to cause massive microtubule damage [59,71,72] and activate kinase pathways such as JNK/SAPK and p34 (cdc2) pathways [73,74,75,76], all of which are associated with paclitaxel-induced apoptosis [57]. It is important to note that apoptosis induced by these pathways is not dependent on mitotic arrest at higher concentrations, suggesting that it may occur in cells at any phase of the cell cycle [55]. This interpretation is consistent with our data that 0.01 µM paclitaxel did not exhibit a significant toxic effect on the morphology of primary DRG non-neuronal cells, whereas the higher concentrations (1 and 10 µM) did.

In the current study, the effect of paclitaxel on the cell viability of primary DRG co-culture by MTT assay was time-dependent and modulated by the presence of neuronal and non-neuronal cells in primary DRG culture. For example, the toxic effects of paclitaxel on the viability of non-neuronal cells alone were apparent earlier, at 24 h post-treatment, while a significant reduction appeared at 72 h after treatment in DRG neuronal cells [17]. However, in primary DRG co-cultures containing neuronal and non-neuronal cells, the effect was present 48 h post-treatment. A possible explanation might be that non-neuronal cells are more susceptible and sensitive to paclitaxel treatment when compared to neurons. As a result, paclitaxel’s effects on non-neuronal cells become more apparent because they are actively growing, whereas post mitotic neurons need longer to respond to cell death [77,78]. Importantly, the effects of paclitaxel on the viability of primary DRG co-culture appeared at 48 h, not 24 h post-treatment, implying that there are cell-cell interactions between neurons and non-neuronal cells and modulating signaling pathways that impact the paclitaxel toxicity in the co-culture. Furthermore, the fate of cells after paclitaxel treatment might be affected by both paclitaxel concentrations and exposure time [51,59].

Neuronal function studies showed that neurons are not the only cell type that contributes to neuronal signaling. In the CNS, non-neuronal cells such as astrocytes, oligodendrocytes, and microglia all play important roles in influencing neuronal activity via interactions between neuronal cells and both glial cells and SGCs [79,80,81,82]. Non-neuronal glial cells and macrophages were shown to play critical roles in neuronal excitability modulation as well as in nutrition, structural, and maintenance functions [83,84]. In addition, they become activated following peripheral nerve injury or chronic inflammation and are involved in controlling neuronal excitability [85]. An interesting structural feature of the sensory ganglia is that the somata of sensory neurons do not form synaptic contacts with one another [86]. Additionally, neuronal cell bodies are enwrapped by SGCs inside the ganglia to form a structural and functional unit [27]. This specific structural arrangement stands for the communication between neurons and SGCs and is a determinant of somatic activity, as recently reported [82]. Changes in communication after injury are critical for understanding the development of abnormal ectopic discharges in somata that influence afferent signaling [28]. As a result, interactions between DRG neurons and glia and the activation of signaling pathways are believed to play an important role in the management of peripheral neuropathy [82].

## 5. Conclusions

Paclitaxel showed a set of toxicological effects on primary DRG non-neuronal cells that included a reduction in cell viability, an increase in cell death, inhibition of cell proliferation, and morphological changes. The effects of paclitaxel on primary DRG non-neuronal cells are concentration- and time- dependent. Given the crucial role of primary DRG non-neuronal cells in supporting DRG neurons and in the development and maintenance of neuropathic pain, the described adverse effects of paclitaxel on DRG non-neuronal cells might have functional consequences for sensory neurons in the DRG and should be considered in the management of peripheral neuropathy. Future research should investigate the potential negative effects of paclitaxel on signaling pathways and interactions between DRG neuronal and non-neuronal cells.

## Data Availability

All datasets generated for this study are included in the article/supplementary material.

## Supplementary Materials

The following supporting information can be downloaded at: https://www.mdpi.com/article/10.3390/toxics11070581/s1, Figure S1: (a) DRG isolation from 6–8 weeks old Wister rats, and (b) extraction and purification of DRG non-neuronal cells by using the density gradient centrifugation method; Figure S2: Representative example of automatic counting of nuclei of non-neuronal cells by the FIJI program for the control group; Figure S3: Effects of different concentrations of paclitaxel on the viability of DRG non-neuronal cells at different investigated time points using the MTT assay; Figure S4: Effects of different concentrations of paclitaxel on the percentage of cytotoxicity of DRG non-neuronal cells at different investigated time windows using the LDH assay; Figure S5: Effects of different concentrations of paclitaxel on the rate of cell proliferation of DRG non-neuronal cells at 24 h, 48 h, and 72 h post-treatment using BrdU assay; Figure S6: Effects of different concentrations of paclitaxel on nuclear morphology (% apoptosis) of DRG non-neuronal cells at 24 h, 48 h, and 72 h post-treatment by DAPI staining; Figure S7: Effects of different concentrations of paclitaxel on the ratio of PI^+^ of DRG non-neuronal cells at 24 h, 48 h, and 72 h post-treatment by propidium iodide assay.
