# Supplementary material for: Time- and Concentration-Dependent Adverse Effects of Paclitaxel on Non-Neuronal Cells in Rat Primary Dorsal Root Ganglia
Source: Toxics. 2023 Jul 4;11(7):581. doi: 10.3390/toxics11070581 (PMC10385404; doi:10.3390/toxics11070581)
Supplement: Supplementary file 1 [file toxics-11-00581-s001.zip › toxics-2439493-supplementary.pdf]

Supplementary information

Time- and Concentration-Dependent Adverse Effects of Paclitaxel on Non-Neuronal Cells in Rat Primary Dorsal Root Ganglia

Amira Elfarnawany[1,2] and Faramarz Dehghani[1*]

[1]Department of Anatomy and Cell Biology, Medical Faculty, Martin Luther University Halle-Wittenberg, Grosse Steinstrasse 52, 06108 Halle (Saale), Germany; amira.elfarnawany@science.tanta.edu.eg

[2] Zoology Department, Faculty of Science, Tanta University, Tanta 31527, Egypt

***Correspondence:** faramarz.dehghani@medizin.uni-halle.de

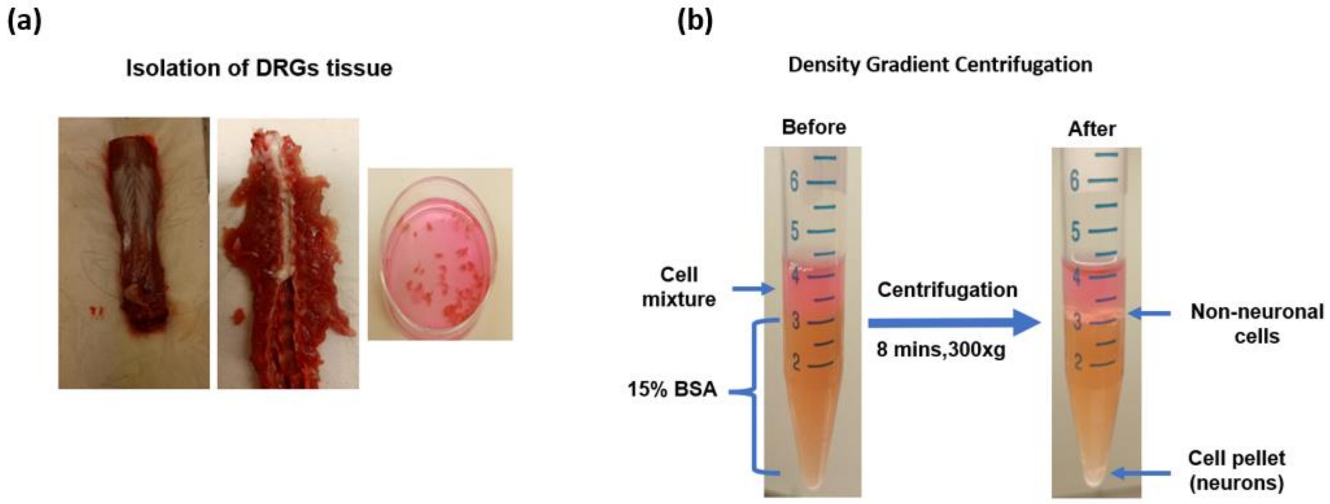

Figure S1. (a) DRGs isolation from 6-8 weeks old Wister rats, and **(b)** extraction and purification of DRG non-neuronal cells by using the density gradient centrifugation method.

(a)

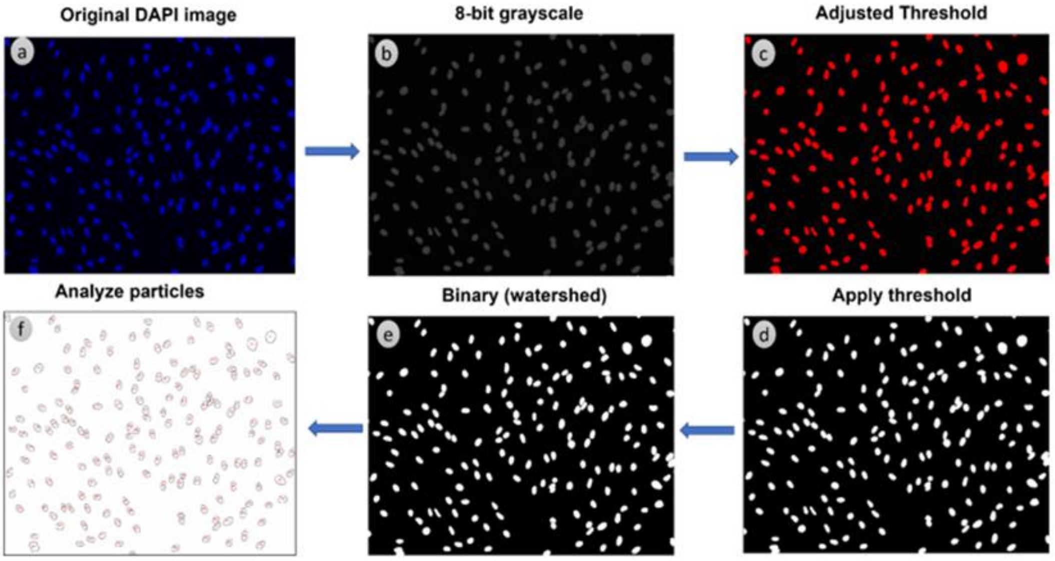

(b)

A	B	C	D	E	F
**Slice**	**Count**	**Total Area**	**Average Size**	**%Area**	**Mean**
100 nM PEA_rep_ch01.tif	**173**	13087252281	75648857.12	8.554	255
100 nM PEA_Series002_ch01.tif	**213**	15721611087	73810380.69	10.276	255
100 nM PEA_Series006_ch01.tif	**150**	10700915261	71339435.07	6.994	255
100 nM PEA_Series009_ch01.tif	**108**	7826254297	72465317.57	5.115	255
100 nM PEA_Series011_ch01.tif	**177**	14087432142	79590012.1	9.208	255
100 nM PEA_Series013_ch01.tif	**186**	15059099381	80962899.9	9.843	255

Figure S2. Representative example for automatic counting of nuclei of non-neuronal cells by FIJI program for the control group. **a)** Step-by-step Demonstration of automatic counting of nuclei (ImageJ). **b**) Results of the different counts of nuclei of non-neuronal cells for six different images for the same control group.

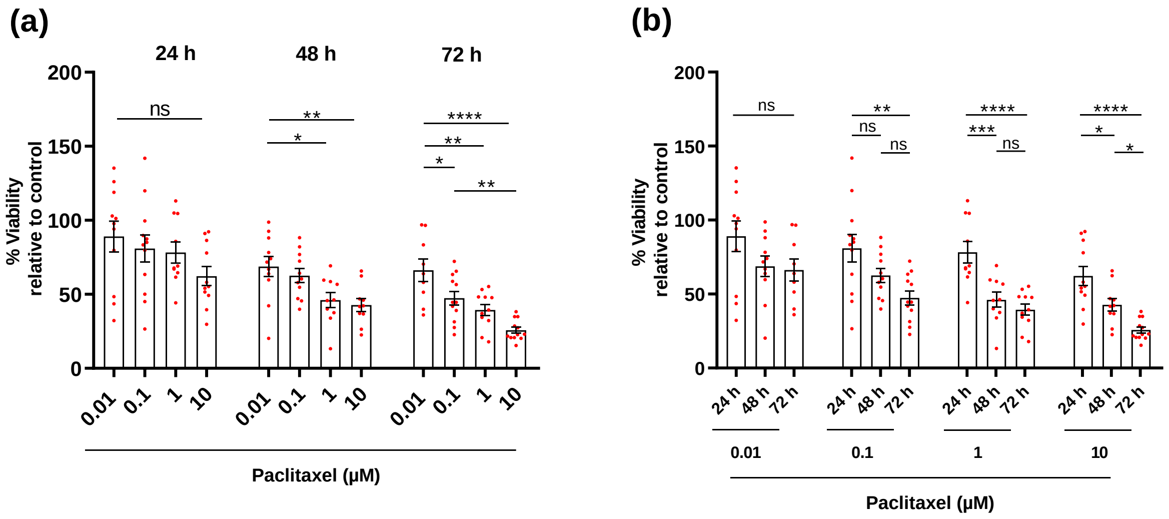

Figure S3. Effects of different concentrations of paclitaxel on the viability of DRG non-neuronal cells at different investigated time points using MTT assay. **(a)** A significant difference was found between different paclitaxel concentrations with a concentration dependency at 48 h, and 72 h post-treatment. **(b)** Time-dependent significant differences were observed for 1 µM, and 10 µM paclitaxel concentrations. Values served as the mean ± SEM of three independent experiments performed in triplicates. The asterisks denote significant results regarding the respective measurement indicated with the bar. The data were analyzed using one-way ANOVA, followed by the Bonferroni post-hock test ($p < 0.05$). * $p < 0.05$, ** $p < 0.01$, *** $p < 0.001$, **** $p < 0.0001$, ns (non-significant).

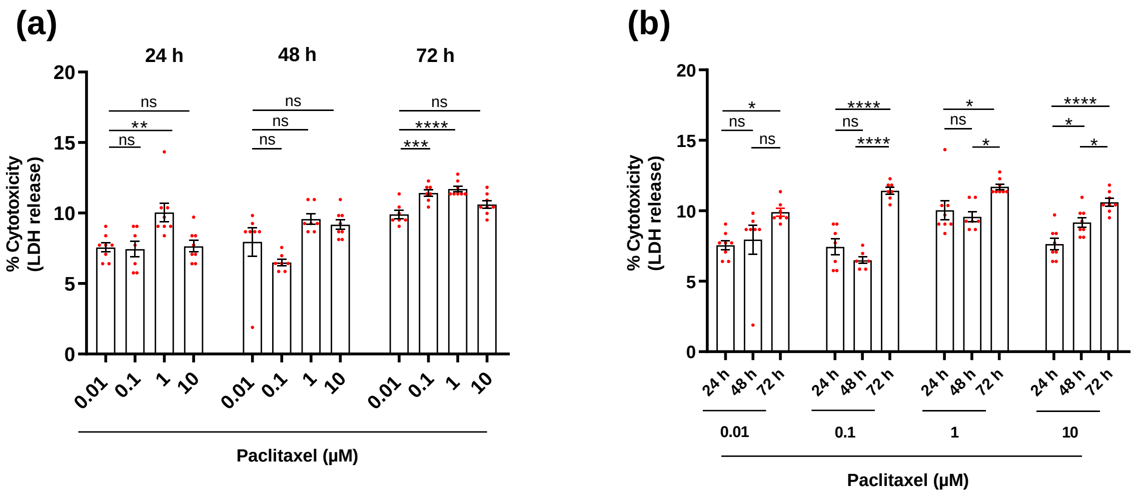

Figure S4. Effects of different concentrations of paclitaxel on the percentage of cytotoxicity of DRG non-neuronal cells at different investigated time windows using LDH assay. **(a)** A significant difference was present between different paclitaxel concentrations, indicating concentration-dependent effects on cell death at 72 h post-treatment. **(b)** for all applied concentrations time-dependent effects were observed for various concentrations of paclitaxel on the cytotoxicity of DRG non-neuronal cells. Values served as the mean ± SEM of three independent experiments and n= 15 replicates. The asterisks denote significant results regarding the respective measurement indicated with the bar. The data were analyzed using one-way ANOVA, followed by the Bonferroni post-hock test ($p < 0.05$). ** $p < 0.01$, *** $p < 0.001$, **** $p < 0.0001$, ns (non-significant).

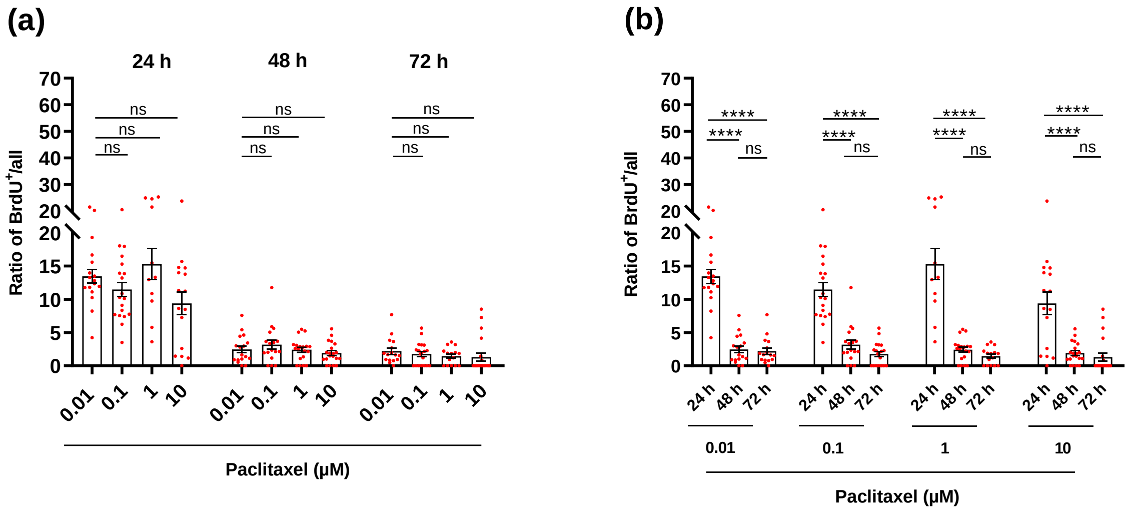

Figure S5. Effects of different concentrations of paclitaxel on the rate of cell proliferation of DRG non-neuronal cells at 24 h, 48 h, and 72 h post-treatment using BrdU assay. **(a)** The bar charts revealed no significant difference between different paclitaxel concentrations, implying that the effects of paclitaxel on cell proliferation are not concentration-dependent at different time points. **(b)** The graph revealed a significant difference between different timelines for all applied concentrations of paclitaxel, indicating that the effects of various concentrations of paclitaxel on the cell proliferation of DRG non-neuronal cells are time-dependent. Values served as the mean ± SEM of three independent experiments and n= 15 replicates. The asterisk denotes statistically significant results for the measurement indicated by the bar. The data were analyzed using one-way ANOVA, followed by the Bonferroni post-hock test (p < 0.05). **** p < 0.0001, ns (non-significant).

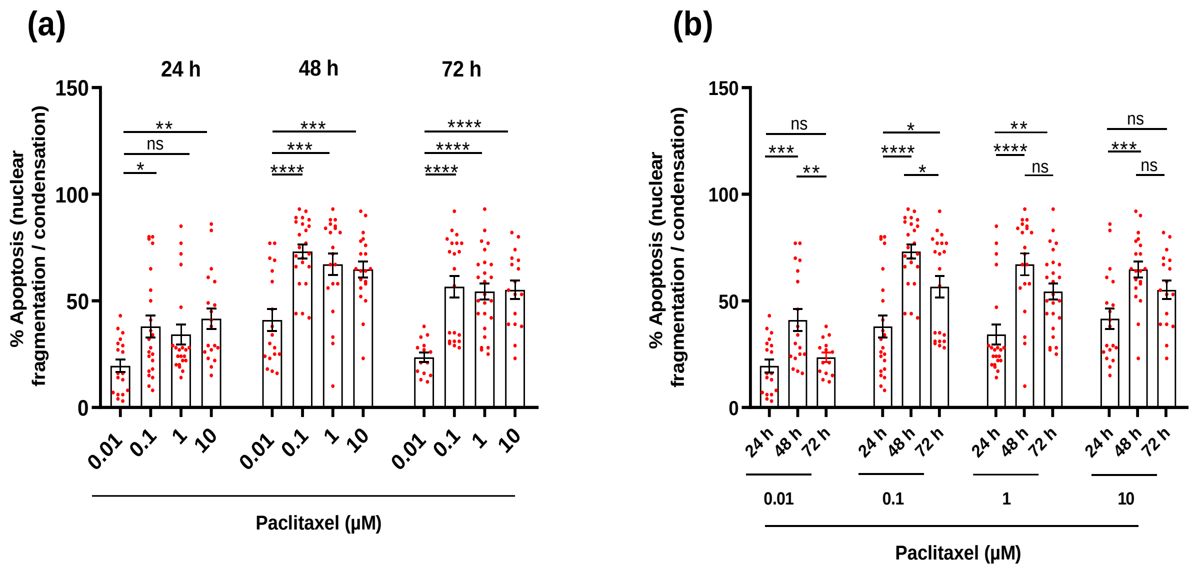

Figure S6. Effects of different concentrations of paclitaxel on nuclear morphology (% apoptosis) of DRG non-neuronal cells at 24 h, 48 h, and 72 h post-treatment by DAPI staining. **(a)** The chart demonstrated a significant difference between different paclitaxel concentrations, implying that the effects of paclitaxel on % of apoptosis are concentration-dependent at different investigated time points. **(b)** The graph showed a significant difference between different timelines specifically between 24 and 48 h of treatment for all concentrations of paclitaxel, indicating that the effects of various concentrations of paclitaxel on the % of apoptosis of DRG non-neuronal cells are time-dependent. Values served as the mean ± SEM of three independent experiments and n= 15 replicates. The asterisk denotes statistically significant results for the measurement indicated by the bar. The data were analyzed using one-way ANOVA, followed by the Bonferroni post-hock test ($p < 0.05$). * $p < 0.05$, ** $p < 0.01$, *** $p < 0.001$, **** $p < 0.0001$, ns (non-significant).

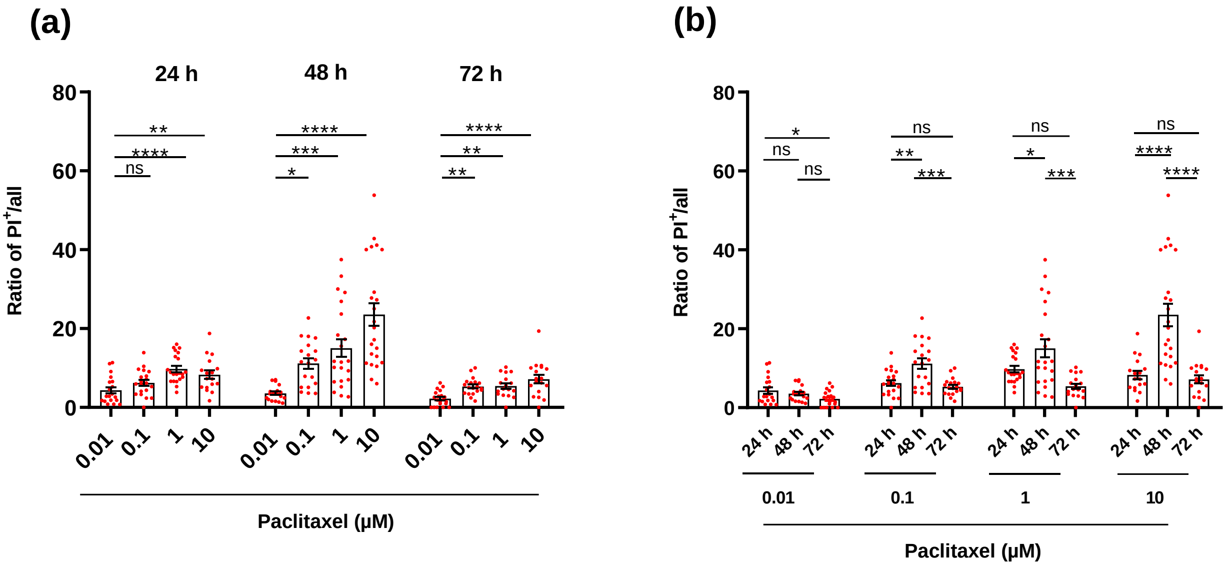

Figure S7. Effects of different concentrations of paclitaxel on the ratio of PI^+ of DRG non-neuronal cells at 24 h, 48 h, and 72 h post-treatment by propidium iodide assay. **(a)** The Bar chart showed a significant difference between different paclitaxel concentrations, implying that the effects of paclitaxel on cell death are concentration-dependent at different investigated time points. **(b)** The graph showed a significant difference between different timelines for all concentrations of paclitaxel except only 0.01 μM paclitaxel, indicating that the effects of various concentrations of paclitaxel on the % of cell death of DRG non-neuronal cells are time dependent. Values served as the mean ± SEM of three independent experiments and n= 15 replicates. The asterisk denotes statistically significant results for the measurement indicated by the bar. The data were analyzed using one-way ANOVA, followed by the Bonferroni post-hock test ($p < 0.05$). * $p < 0.05$, ** $p < 0.01$, *** $p < 0.001$, **** $p < 0.0001$, ns (non-significant).
